# A composite score combining procalcitonin, C-reactive protein and temperature has a high positive predictive value for the diagnosis of intensive care-acquired infections

**DOI:** 10.1186/1471-2334-13-159

**Published:** 2013-04-02

**Authors:** Laurent Robriquet, Caroline Séjourné, Eric Kipnis, Michele D’herbomez, François Fourrier

**Affiliations:** 1 Service de Réanimation Polyvalente, Centre Hospitalier Universitaire de Lille, 59000 Lille, France; 2 Service de Réanimation Chirurgicale, Centre Hospitalier Universitaire de Lille, 59000 Lille, France; 3 Service de Médecine Nucléaire, Centre Hospitalier Universitaire de Lille, 59000 Lille, France; 4 Service de Réanimation Polyvalente, Hôpital Roger Salengro - Centre Hospitalier Universitaire de Lille, Rue Emile Laine, 59037 Lille, Cedex France

**Keywords:** Nosocomial infection, Procalcitonin, C-reactive protein, Intensive care unit

## Abstract

**Background:**

Nosocomial infection diagnosis in the intensive care unit (ICU) remains a challenge. We compared routine measurements of procalcitonin (PCT), C-reactive protein (CRP), white blood cell count (WBC) and temperature in the detection of ICU-acquired infections.

**Method:**

Prospective observational cohort study in a University hospital Medicosurgical ICU. All patients admitted to the ICU ≥ 5 days (n = 141) were included into two groups, either infected (documented infection, n = 25) or non-infected (discharged from the ICU without diagnosis of infection, n = 88).

**Results:**

PCT, CRP, WBC and temperature progression from day −4 (D-4) to day 0 (D0) (day of infection diagnosis or ICU discharge) was analysed. Differences (Δ) were calculated as D0 levels minus the lowest preceding value. D0 PCT and CRP were significantly increased in infected compared to non-infected patients (median, 1^st^ and 3^rd^ quartiles): 3.6 ng/mL (0.92-25) for PCT, 173 mg/L (126–188) for CRP versus 0.02 ng/mL (0.1-0.9) and 57 mg/mL (31–105) respectively (p < 0.0001). In multivariate analysis, D0 temperature > 38.6°C, PCT > 1.86 ng/mL, and CRP > 88 mg/L, performed well (AUCs of 0.88, 0.84, and 0.88 respectively). The sensitivity/specificity profiles of each marker (76%/94% for temperature, 68%/91% for PCT, and 92%/70% for CRP) led to a composite score (0.068 × D0 PCT + 0.005 × D0 CRP + 0.7 × temperature) more highly specific than each component (AUC of 0.90 and sensitivity/specificity of 80%/97%).

**Conclusion:**

Combining CRP, PCT and temperature is an approach which may increase of nosocomial infection detection in the ICU.

## Background

Early identification of nosocomial infections is crucial to therapeutic decision-making, allowing both early antimicrobial therapy and source eradication which determine outcome [1]. However, early discrimination between sepsis due to nosocomial infection (NI) and systemic inflammatory response syndrome (SIRS) is challenging in clinical practice. Indeed, in early sepsis, clinical signs related to the focus of infection may be minimal or obscured by non-specific symptoms of SIRS common to various non-infectious inflammatory conditions. Definitive diagnosis of infection is defined as the identification of pathogens in a biological sample from a normally sterile tissue or fluid [2]. However, this definition still relies on clinical suspicion of infection since colonizing pathogens may be present without leading to the pathogen-related damage which defines infection [2]. The reliance of diagnosis on clinical suspicion rather than identification of pathogens is particularly true for nosocomial infection which occurs in hospitalized patients, increasingly colonized by various pathogens over time. This absence of any “gold-standard” of infection (with some exceptions such as bacterial meningitis, fungemia etc.…) has resulted in a decades-long quest for biomarkers of infection capable of assisting or even redefining the diagnosis of infection.

In the ICU, early, sensitive, and specific laboratory tests would be crucial to guide clinicians in identifying infected patients who could benefit from prompt empirical antibiotic therapy and to avoid unnecessary antibiotic treatments.

The aim of our prospective cohort study was to test the hypothesis that inflammatory biomarkers procalcitonine (PCT) and C-reactive protein (CRP), in addition to routine biomarkers such as white blood-cell count (WBC) and clinical markers such as fever, could assist in the early identification of patients with ICU-acquired nosocomial infection.

## Methods

### Study design and inclusion criteria

This prospective observational study was conducted in a 16-bed medico-surgical university ICU from July 2007 to March 2008. Ethics committee approval was obtained from the French Society of Intensive Care Medicine (SRLF-CE-157) for our trial and written informed consent was obtained from each patient or designated surrogate. All patients who were aged > 18 years admitted the ICU for more than 4 days were eligible. Only the first admission to the ICU was recorded. Patients were excluded if they were pregnant, already included in another trial, or undergoing care limitation or withdrawal.

### Definitions

Clinically suspected VAP was defined as a new or persistent pulmonary infiltrate on the chest radiograph, with at least two of the following criteria: (i) temperature > 38°C or < 36°C; (ii) WBC > 10 or < 4 × 10^3^/mm^3^; (iii) purulent tracheal aspirate [3,4]. Microbiological confirmation was defined by the presence of at least one potentially pathogenic microorganism in respiratory samples according to predefined thresholds (bronchoalveolar lavage fluid samples yielding 10^4^ CFU/mL or tracheal aspirates yielding 10^6^ CFU/mL).

Bloodstream infection (BSI) was defined as the occurrence of infection associated with one or more positive blood culture results unrelated to an infection incubating at ICU admission. In case of coagulase-negative Staphylococci, two positive blood cultures on separate occasions within a 48-hrs period, and confirmation of clinical significance by the attending intensivist were required for diagnosis of bacteremia [5]. All Other ICU-acquired infections were defined according to the modified Centre for Disease Control and Prevention criteria [6]. Blood cultures were routinely performed when the patients' temperature was ≥ 38.5°C or < 36°C or when infection was suspected on clinical grounds.

SIRS was diagnosed in the presence of more than one of the following clinical findings: (i) body temperature higher than 38°C or lower than 36°C, (ii) heart rate higher than 90 beats per min, (iii) hyperventilation evidenced by respiratory rate higher than 20/min or arterial partial pressure of carbon dioxide (PaCO_2_) lower than 32 mmHg, (iv) WBC higher than 12 000 cells/μL or lower than 4 000 cells/μL.

### Study design and data collection

We followed a previously described design and protocol for data collection [7]. Briefly, “non-infected” patients had no bacteriological or clinical signs of nosocomial infections and “infected” patients were those with ICU-acquired, therefore nosocomial, infection. For analysis, only the first episode of nosocomial infection was considered. Day 0 (D0) was defined as the day of nosocomial infection diagnosis for infected patients or the day of ICU discharge for non-infected patients. Daily values of PCT, CRP, temperature, WBC and SOFA score from four days prior to day 0 (D-4) up to day 0 (D0) ware collected and analysed comparing infected and non-infected patients. For comparisons, daily values on D0 and D-4 were considered as well as biomarker variations over time (ΔPCT and ΔCRP) calculated as the difference between D0 value minus the lowest PCT and CRP value over the previous 4 days.

Collected data included age, gender, admission diagnosis, systemic inflammatory response syndrome (SIRS), Simplified Acute Physiology Score II (SAPS II), Sequential Organ Failure Assessment (SOFA) score, central vein catheterization and dwell time, mechanical ventilation and duration, anti-infectious treatment, ICU length of stay and patient outcome [8]. PCT, CRP and WBC were measured at admission and daily until ICU discharge or death in all patients. Body temperature was measured every three hours and daily lowest and highest values were recorded. Patients were assessed daily for clinical evidence of infection, and appropriate samples for bacteriological cultures were collected whenever infection was suspected.

### Biomarkers

Blood samples were obtained from an arterial line upon admission and subsequently daily at 07:00. PCT was measured by time-Resolved Amplified Cryptase Emission technology in a Kryptor® analyser (Brahms Diagnostica, Berlin, Germany). The sensitivity of the assay was 0.05 ng/mL. CRP was measured by an immunoturbidimetric assay (Advia 2400, Bayer Diagnostics, Tarrytown, NY). WBC was quantified by the hospital hematology laboratory using an automated cell analyser (XE 20100, Sysmex, Japan).

### Statistical analysis

All results are presented as number (percentage) for categorical variables and median and 25^th^/75^th^ percentiles for quantitative variables (data non-normally distributed). The Kolmogorov-Smirnov test was used to assess sample distributions. Continuous variables were compared using the Mann–Whitney (2-group comparison) or Kruskall Wallis (multiple-group comparison) non-parametric tests. Categorical variables were compared with the χ^2^ or the Fisher’s exact test, as appropriate. All p values were two-tailed. Statistical significance was defined as p < 0.05. Time-dependent analysis of different variables was performed with a general linear model, univariate repeated-measures analysis using a split-plot design approach. We studied PCT, CRP, temperature, WBC at D0 and D-4, the ΔPCT and ΔCRP in a univariate analysis. Multivariate regression logistic analysis was used to determine values independently associated with ICU-acquired infection and Odds ratio (OR) with 95% confidence interval (CI) were calculated. Receiver–operating characteristic (ROC) curves and the areas under the curve (AUC) were determined for PCT, CRP, temperature, WBC at D0 and for the ΔPCT and ΔCRP. The AUC values are reported with the 95% confidence interval. In medical practice, a diagnostic test with an AUC < 0.75 would be regarded as non contributive. Sensitivities, specificities, positive predictive values and negative predictive values were calculated from cross-tabulations. A composite score was determined using the best values predicting nosocomial infection. Statistical analyses were performed using the SAS software version 8.2 (SAS Institute Inc., Cary, NC, USA).

## Results

During the study period, 267 patients were admitted to our ICU. One hundred and twenty-six patients were excluded (24 obstetrical patients, 8 patients under 18 years of age, 81 patients who stayed less than 4 days, and 4 patients who had treatment limited or withdrawn). One hundred and forty-one patients were included in the study, 116 non-infected among which 88 were non-infected and discharged alive from the ICU. Twenty-five patients were diagnosed with infections acquired in the ICU.

**Figure 1:**
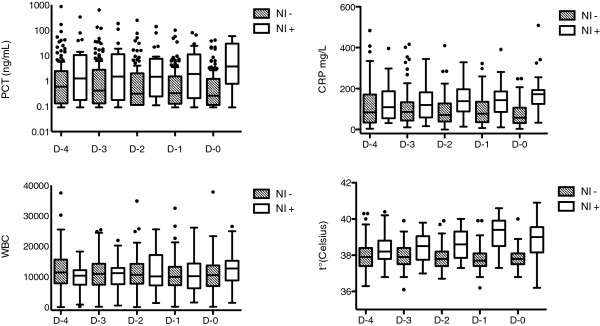
**Dynamics of serum PCT and CRP concentrations, WBC and temperature in patients-acquired ICU infection and non-infected patients from D-4 to D0.** NI: nosocomial Infection. Data are presented as box plot. *: p < 0.05.

Age, sex distribution and severity of illness upon ICU admission were not statistically different between patients with ICU-acquired infection (n = 25) and non-infected (n = 88) (Table 1). Concerning ICU-acquired infections, 11 BSI, 9 VAP, 2 catheter-related infections and 3 fungemia were recorded. Nosocomial infection occurred 11 days (8–14) after ICU admission. The median number of days without antibiotics before nosocomial infection diagnosis was 5 days (3.5-5.5) and the median of duration of mechanical ventilation and length of ICU stay were significantly higher in patients with ICU-acquired infection compared to non-infected patients: 25 days (15–33) vs. 10 days (5–14), p < 0.001; and 29 days (22–39) vs. 12 days (7–18), p < 0.0001. SIRS criteria were present at D0 in 100% of infected patients vs. 74% of non-infected patients (p = 0.01). The median of SOFA score was significantly higher in infected compared to non-infected patients: 8 (6–12) vs. 1 (0–3.5) at D0 and 7 (5–9) vs. 3 (2–6) at D-4, p < 0.001. Time-dependent analysis of both PCT and CRP during the 4 days prior to diagnosis of nosocomial infection showed a significant increase in infected patients whereas PCT and CRP levels decreased over the time in the non-infected group (Figure 1). PCT and CRP D0 median concentrations were both significantly increased in the infected group compared non-infected group, 3.6 ng/mL (0.92-25) vs. 0.2 ng/mL (0.1-0.9) and 173 mg/L (126–188) vs. 57 mg/L (31–101), p < 0.001, respectively. A similar result was obtained for maximal daily temperature: the median value of maximal daily temperature at D0 was significantly increased in the infected group compared to the non-infected one: 39°C (38.2-39.5) versus 37.8°C (37.5–38.1), p < 0.001. WBC values from D-4 to D0 were not significantly different between infected and non-infected patients. Dynamic data on PCT, CRP, WBC and temperature from D-4 to D0 are shown in Figure 1.

**Table 1:** Characteristics of the 113 studied patients during ICU stay

**Characteristics of patient population**	**NI - (88)**	**NI + (25)**	**p**
Age (years)	55 (45–65)	55 (47–59)	0.34
Sex (male/female)	48/40	17/8	0.38
SAPS II score at admission	45 (31–55)	50 (38–57)	0.43
Admission diagnosis, n (%)			0.07
Acute respiratory failure	26 (30)	5 (20)	
Neurologic failure	22 (25)	5 (20)	
Cardiovascular failure	16 (18)	3 (12)	
Polytrauma	10 (11)	6 (24)	
Post operative	3 (3)	5 (20)	
Miscellaneous	11 (13)	1 (4)	
Site of infection, n (%)			
Bacteremia		11 (44)	
Pneumonia		9 (36)	
Catheter related infection		2 (8)	
Candidemia		3 (12)	
Delay of NI, days		11 (8–14)	
Number of days without antibiotics before NI		5 (3.5-5.5)	
Central venous catheter, n (%)	60 (68)	25 (100)	0.005
Duration of central venous catheter, days	11 (8–17)	22 (14–35)	0.001
MV, n (%)	58 (66)	24 (96)	0.02
Duration of MV, days	10 (5–14)	25 (15–33)	0.0007
Length of ICU stay, days	12 (7–18)	29 (22–39)	< 0.0001

Data are presented as n (%) and median values (lower and upper quartiles). NI: Nosocomial Infection. SAPS: Simplified Acute Physiologic Score. MV: mechanical ventilation. ICU: Intensive Care Unit.

Variables included in the univariate logistic regression model for identification of patients with ICU-acquired infection were: PCT, CRP, WBC, and maximal daily temperature at both D-4 and D0, ΔPCT and ΔCRP. Results are shown in Table 2. No D-4 parameters were significantly different between infected and non-infected patients and were not studied further. All D0 parameters, except D0 WBC, were significantly different between infected and non-infected patients and D0 WBC was included in subsequent analyses for comparison. Analysis of the time course, as expressed by ΔPCT and ΔCRP levels, showed that the median ΔPCT in patients with ICU-acquired infection was 1.6 ng/mL (0.06-7.01) vs. 0 (0–0) in non-infected patients (p < 0.0001) and ΔCRP was 75 mg/L (37–130) vs 0.5 mg/L (0–21), p < 0.0001. Stepwise logistic regression independently identified three factors in early identification of ICU-acquired infections: PCT level at D0 (OR: 1.09; 95% CI: 1.03-1.16), maximal daily temperature at D0 (OR: 3.07; 95% CI: 1.53-6.14) and ΔCRP (OR: 1.02; 95% CI: 1–1.03) (Table 3). Although ΔPCT was not significant in multivariate analysis, we included it in subsequent analyses for comparison.

**Table 2:** Results of univariate analysis

	**NI - (88)**	**NI + (25)**	**p**
PCT D-4 (ng/mL)	0.44 (0.12–1.82)	1.25 (0.18–9.2)	0.18
PCT D0 (ng/mL)	0.2 (0.1-0.9)	3.6 (0.92–25)	< 0.0001
CRP D-4 (mg/L)	84 (34–164)	109 (59–179)	0.18
CRP D0 (mg/L)	57 (31–105)	173 (126–188)	< 0.0001
WBC D-4 (cells/mm^3^)	11430 (7900–15600)	10320 (7445–11955)	0.28
WBC D0 (cells/mm^3^)	10540 (7115–13512)	12680 (8800–13500)	0.08
Temperature D-4 (°C)	37.9 (37.4–38.4)	38.2 (37.8–38.7)	0.08
Temperature D0 (°C)	37.8 (37.5–38.1)	39 (38.2–39.5)	< 0.0001
ΔPCT (ng/mL)	0 (0–0)	1.6 (0,06–7.01)	< 0.0001
ΔCRP (mg/L)	0.5 (0–21)	75 (37–130)	< 0.0001

Data are presented as median values (lower and upper quartiles). NI: Nosocomial Infection. *: p < 0.05.

**Table 3:** Results of multivariable logistic regression model

	**Odds ratio**	**95% confidence interval**
PCT D0 (ng/mL)	1.09	1.03 – 1.16
Température D0 (°C)	3.07	1.53 – 6.14
ΔCRP (mg/L)	1.02	1 – 1.03

Variations per unit of measurement.

**Figure 2:**
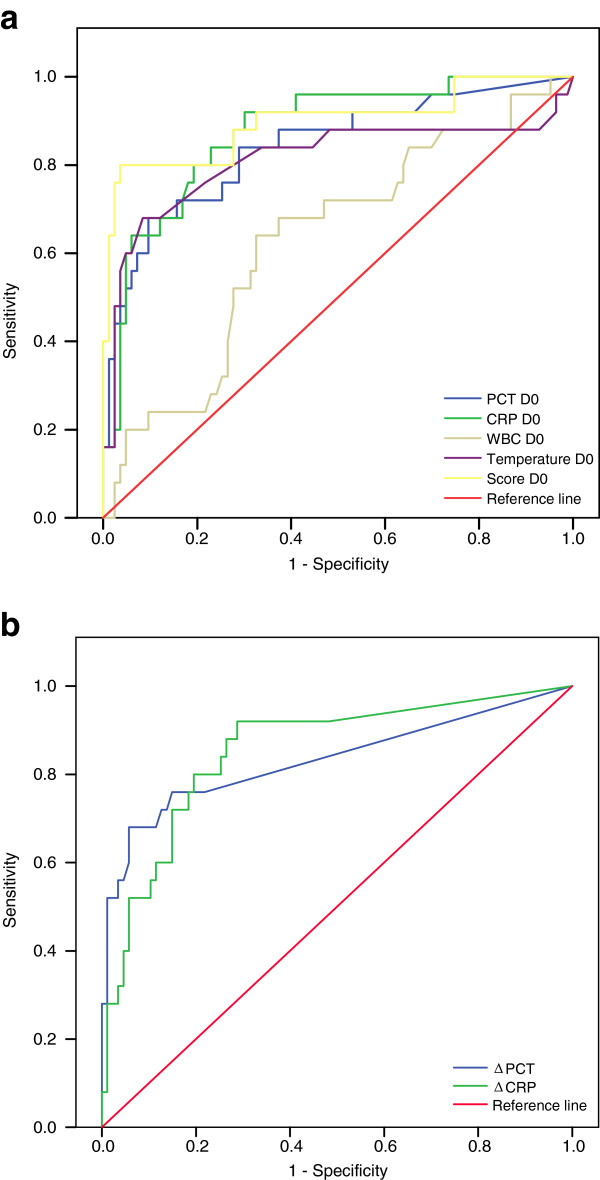
Receiver operating characteristic (ROC) curves comparing markers’ ability to detect ICU-acquired infections, a) ROC curves comparing PCT, CRP, WBC, temperature, and composite score, b) ROC curves comparing ΔPCT and ΔCRP for detection of ICU-acquired infections.

ROC curves were plotted to compare the detection of nosocomial infection by PCT, CRP, WBC, and temperature at D0. The AUC of D0 WBC was poor of only 0.62. However the AUC for D0 temperature was 0.88 and temperature at D0 > 38.6°C resulted in a sensitivity/specificity of 76%/94%. AUCs for PCT and CRP D0 were 0.84 and 0.88 respectively, and were not significantly different (p = 0.67) indicating that the tests globally performed similarly in discriminating nosocomial infection. However, using the best cut-offs for PCT and CRP D0 values which were 1.86 ng/mL and 88 mg/L respectively, the resulting sensitivity/specificity were inverted: low specificity/high sensitivity of 68%/91% for PCT and high sensitivity/low specificity of 92%/70% for CRP. The best cut-off values and AUCs for all clinical and biological makers are shown in Table 4 and ROC curves are represented in Figure 2. The combination of the three markers with AUCs > 0,8 over their respective optimal thresholds, D0 PCT > 1.86 ng/mL, D0 CRP > 88 mg/L and D0 temperature > 38°C, was present in 56% of infected patients compared to 1% of non-infected patients. A composite marker was therefore constructed, combining PCT, CRP levels and temperature at D0 (score = 0.068 × D0 PCT + 0.005 × D0 CRP + 0.7 × Temperature D0). The AUC for this composite score was 0.90, the best cut-off for the composite score was 28, resulting in a sensitivity/specificity of 80%/97%. Similarly to D0 values, although AUCs for ΔCRP and ΔPCT were not statistically different (0,83 and 0,85), ΔPCT, with a > 0.49 ng/mL threshold resulted in a low sensitivity/high specificity of 68%/94% while ΔCRP, with a threshold > 12 mg/L, resulted in high sensitivity/low specificity of 92%/71% for identifying nosocomial infection.

**Table 4:** Diagnosis accuracy of serum PCT, CRP, WBC, body temperature, ΔCRP and ΔPCT for diagnosis of ICU-acquired infection

	**AUC**	**Cut-off level**	**Sensitivity (%)**	**Specificity (%)**	**PPV**	**NPV**
PCT D0	0.84	1.86 ng/mL	68	91	68	91
CRP D0	0.88	88 mg/L	92	70	47	97
WBC D0	0.62	12120/mm^3^	64	68	36	87
Temperature D0	0.88	38.6°C	76	94	79	94
Composite score D0	0.90	28	80	97	87	94
ΔPCT	0.83	0.49 ng/mL	68	94	77	91
ΔCRP	0.85	12 mg/L	92	71	48	97

Data are presented as % unless otherwise stated. AUC: area under the receiver-operating-characteristic curve; NPV: negative predictive value; PPV: positive predictive value.

## Discussion

By studying simple and routinely used clinical and biological markers of infection in patients with confirmed nosocomial infection compared to non-infected ICU patients we determined that three parameters, temperature > 38.6°C, PCT > 1.86 ng/mL, and CRP > 88 mg/L, could perform well in discriminating infected from non-infected patients (AUCs of 0.88, 0.84, and 0.88 respectively). The complementary sensitivity/specificity profiles of each marker (76%/94% for fever, 68%/91% for PCT, and 92%/70% for CRP) allowed the construction of a composite score (score = 0.068 × D0 PCT + 0.005 × D0 CRP + 0.7 × temperature) more discriminating and highly specific than each single component (AUC of 0.90 and sensitivity of 97%).

Given the absence of any gold standard of infection we used the same methodology as Povoa et al. [7] and distinguished between patients with a confirmed diagnosis of noscomial infection, with all the limitations inherent to diagnosis of infection in the ICU, and patients discharged from the ICU without being treated by antimicrobials and therefore very unlikely to be infected.

Although one of the most frequently measured parameters in the ICU setting and non invasive, body temperature remains a poor indicator of infection [9]. Temperature can be influenced by a number of non-infectious factors, such as non-infectious causes of fever and antipyretic therapy [10]. Nevertheless, in the present study, temperature appeared to perform reasonably well for identifying nosocomial infection, with an AUC of 0.88. The best temperature cut-off value was 38.6°C, resulting in a sensitivity-specificity of 76%/94%.

Our data support the view of some authors that leucocyte count has little value in discriminating patients with nosocomial infection [7]. In our study, AUC of WBC was 0.62, indicating that WBC was close to the line of non discrimination.

PCT is secreted as part of the systemic inflammatory response to infection and serum values are greatly based on the type and severity of infection. Interpretation of the literature is further complicated by frequent discrepancies or variations in the choice of the cut-off value of PCT, etiologies of infection, severity of infection, and study populations [11,12]. Serum values of PCT vary greatly based on the type and severity of infection. The highest PCT concentrations have been reported in patients with septic shock, and patients with severe sepsis had significantly higher PCT levels than patients with sepsis or SIRS [13].

In the present study, we used an ultrasensitive assay for PCT, capable of measuring low levels to identify even “subclinical” inflammatory states before the development of clinically evident sepsis. The best cut-off value of PCT for identifying ICU-acquired infection was 1.86 ng/mL, with high specificity (91%) but low sensitivity (68%).

Few studies have analysed the behaviour of PCT in nosocomial ICU-acquired infection. In cardiac surgery patients, PCT measurement was found a reliable marker for diagnosis of infection: with a cut-off of 1 ng/mL allowing a sensitivity of 85% and a specificity of 95% [14]. In another study, PCT was useful in the diagnosis of VAP with a cuff-off value of 3.9 ng/mL allowing a specificity of 100% at the cost of a sensitivity of 41% [15].

In a recent study assessing PCT monitoring in the early diagnosis of nosocomial infection, PCT at D0 was the best predictor of proven infection. A cut-off value of 0.44 ng/mL provided sensitivity and specificity of 65.2% and 83%, respectively for discriminating patients with proven nosocomial infection from clinically suspected but non-proven nosocomial infection [16].

Several reports suggested that PCT should replace CRP as a marker of infection in the ICU setting [17,18]. In certain situations, especially to differentiate bacteremic from non-bacteremic infections, PCT was reported to be superior to CRP [19]. A cut-off value of 0.4 ng/mL was associated with a negative predictive value of 98%. However, well-designed studies have shown that PCT is neither a better nor an earlier diagnostic marker of infection than CRP [20-22].

In a previous meta-analysis performed by Tang et al., PCT could not reliably differentiate sepsis from other non-infectious causes of systemic inflammatory response syndrome in critically ill adult patients [23]. On the other hand, in another review, PCT concentration was found to be better than CRP for diagnosis of bacterial infection [12]. However, this review included studies across a wide range of age group, clinical setting and spectrum diseases: 46% were paediatric patients and 57% did not have SIRS. Additionally, in some clinical situations of infectious origin commonly found in ICU, PCT can be normal or even undetectable early course of infections [24], localised infections [25], or subacute endocarditis [19,26,27].

CRP is an acute-phase protein, member of the pentraxin family of proteins, whose hepatic synthesis is triggered by cytokine release due to any cause of inflammation, infectious or not. In the present study, CRP D0 had the highest AUC (0.88) and a cut-off of 88 mg/L had a sensitivity of 92% and a specificity of 70% to identify patients with ICU-acquired infection. Povoa et al. studied the role of CRP to detect infections in critically ill patients [28]. In that study, the combination of CRP > 87 mg/L and body temperature > 38.2°C was associated with infection diagnosis with a specificity of 100%. A study using the methodology we based our study upon, found that daily CRP monitoring could be used as a marker of infection prediction [7]. Patients presenting maximum daily CRP variation > 4.1 mg/dL and a CRP level > 87 mg/L had an 88% risk of infection.

As all preceding studies we have confirmed that no single parameter or biomarker can reliably assist the clinician in diagnosing infection in the ICU. This is most probably due to: the lack of any possible gold standard for the diagnosis of infection and the extreme heterogeneity of infection in the ICU as to causal pathogen, underlying diseases, host-response, therapy, evolution and outcome. Facing these problems, our subsequent combination of diagnostic makers appears a useful approach to improve the accuracy in diagnosing nosocomial infection in ICU patients. Our results showed that combining PCT, CRP and temperature D0 was discriminant (highest AUC = 0.90) and highly specific (specificity of 97%). We found in our study a complementarity of CRP and PCT, with low specificity/high sensitivity for PCT and high sensitivity/low specificity for CRP. Such a combined clinical and multibiomarker approach for prediction has been proposed in another highly heterogenous complex disease in the ICU: adult respiratory distress syndrome (ARDS). Ware et al., studying the prediction of mortality in the ARDS cohort from the NHLBI studies, found that a combination of biomarkers and clinical predictors was superior to clinical predictors or biomarkers alone [29].

Some limitations of the present investigation should be noted. Our findings are based on a single centre study, one should be cautious in or extrapolating these data. Our findings cannot be generalised to specific diseases (pancreatitis, burns) or settings (cardiovascular surgical patients, neonatal/paediatric patients). Due to our study design, our findings might be only applicable to patients with late-onset nosocomial infection. Likewise, our focus on late-onset nosocomial infection led to a small number (17%) of documented infections which could also be a limitation.

It must also be acknowledged that CRP at D0 had a negative predictive value higher than our composite score (97% vs. 94%), showing that low CRP, under the threshold of 88 mg/L could assist clinicians in eliminating the diagnosis of nosocomial infection. However, the composite score had by far the highest positive predictive value (87% vs. 47%), suggesting that it could best be used in encouraging clinicians to initiate antimicrobial therapy when faced with a suspected diagnosis of nosocomial infection and a CRP over 88 mg/L.

## Conclusion

The sequential measurements of serum PCT and CRP might be reliable and complementary biomarkers in early identification of ICU-acquired nosocomial infection. Combining CRP and PCT levels with temperature is an original approach which may increase diagnostic specificity. More prospective and large-scale studies are required to define the best approach.

## Abbreviations

ICU: Intensive Care Unit; SIRS: Systemic inflammatory response syndrome; PCT: Procalcitonin; CRP: C-reactive protein; WBC: White blood cell count (WBC); VAP: Ventilator-associated pneumonia; SOFA: Sequential Organ Failure Assessment score.

## Competing interests

The authors declare that they have no competing interests.

## Authors’ contributions

RL collected and analyzed the data, developed the study design and was the principal writer of the manuscript. SC collected and analyzed the data. KE was involved in the drafting of the manuscript, the discussion and the results. DM was involved in the construction of the protocol. FF critically reviewed the manuscript and was involved because of his expertise in the field. All authors read and approved the final version of the manuscript.

## Pre-publication history

The pre-publication history for this paper can be accessed here:

http://www.biomedcentral.com/1471-2334/13/159/prepub
